# Supplementary material for: Structure and Physical Properties of Cardamonin: A Spectroscopic and Computational Approach
Source: Molecules. 2020 Sep 6;25(18):4070. doi: 10.3390/molecules25184070 (PMC7570488; doi:10.3390/molecules25184070)
Supplement: Supplementary file 1 [file molecules-25-04070-s001.zip › Supplementary Materials/Supporting Materials.docx]

Supporting Materials

Structure and physical properties of cardamonin: a spectroscopic and computational approach

Iwona Budziak ^1^, Marta Arczewska ^2.^*, and Daniel M. Kamiński ^3.^*

^1^ Department of Chemistry. University of Life Sciences in Lublin. Akademicka 15. 20-950 Lublin. Poland; [iwona.budziak@up.lublin.pl](mailto:iwona.budziak@up.lublin.pl)

^2^ Department of Biophysics. University of Life Sciences in Lublin. Akademicka 13. 20-950 Lublin. Poland; [marta.arczewska@up.lublin.pl](mailto:marta.arczewska@up.lublin.pl)

^3^ Department of Chemistry. Maria Curie-Sklodowska University in Lublin. pl. Marii Curie-Skłodowskiej 2. 20-031 Lublin. Poland; [daniel.kaminski@poczta.umcs.lublin.pl](mailto:daniel.kaminski@poczta.umcs.lublin.pl)

***** Correspondence: [marta.arczewska@up.lublin.pl](mailto:marta.arczewska@up.lublin.pl) (M.A.); [daniel.kaminski@poczta.umcs.lublin.pl](mailto:daniel.kaminski@poczta.umcs.lublin.pl) (D.M.K); Tel./Fax: +(48-81)445-69-05 (M.A.); Tel./Fax: +(48-81)537-56-44 (D.M.K.)

**Table S1.** Results of the DFT calculations of electronic transition within the range of 300 nm to 500 nm (E. in eV) and oscillator strengths for **CA** species.

| **CA** | | | | |
| --- | --- | --- | --- | --- |
| **Species** | **Exc. state** | **E [eV]** | **λ[nm]** | ***f*** |
| **Dimer I** | 1 | 2.9222 | 424.28 | 0.0000 |
|  | 2 | 2.9898 | 414.69 | 0.0000 |
|  | 3 | 3.0361 | 408.37 | 0.0188 |
|  | 4 | 3.0790 | 402.68 | 0.0813 |
|  | 5 | 3.1337 | 395.64 | 0.3165 |
|  | 6 | 3.1652 | 391.71 | 0.0000 |
|  | 7 | 3.4437 | 360.03 | 1.1084 |
|  | 8 | 3.5704 | 347.25 | 0.0000 |
|  | 9 | 3.6361 | 340.99 | 0.0041 |
|  |  |  |  |  |
| **Dimer II** | 1 | 3.1642 | 391.84 | 0.0015 |
|  | 2 | 3.1664 | 391.56 | 0.0535 |
|  | 3 | 3.2695 | 379.22 | 0.0682 |
|  | 4 | 3.2841 | 377.52 | 0.0221 |
|  | 5 | 3.4629 | 358.03 | 0.0006 |
|  | 6 | 3.4906 | 355.19 | 0.0195 |
|  | 7 | 3.5599 | 348.28 | 0.0462 |
|  | 8 | 3.6172 | 342.77 | 0.0427 |
|  | 9 | 3.6234 | 342.17 | 0.5783 |
|  |  |  |  |  |
| **Dimer III** | 1 | 2.9598 | 418.89 | 0.0085 |
|  | 2 | 3.0852 | 401.87 | 0.0051 |
|  | 3 | 3.1045 | 399.36 | 0.0115 |
|  | 4 | 3.1798 | 389.91 | 0.0067 |
|  | 5 | 3.2682 | 379.37 | 0.0404 |
|  | 6 | 3.3168 | 373.80 | 0.0145 |
|  | 7 | 3.3921 | 365.51 | 0.0197 |
|  | 8 | 3.5279 | 351.44 | 0.6607 |
|  | 9 | 3.5818 | 346.16 | 0.3099 |
|  |  |  |  |  |
| **Dimer IV** | 1 | 3.0155 | 411.16 | 0.0107 |
|  | 2 | 3.1113 | 398.49 | 0.0277 |
|  | 3 | 3.1635 | 391.92 | 0.0219 |
|  | 4 | 3.2129 | 385.89 | 0.0522 |
|  | 5 | 3.3084 | 374.76 | 0.0400 |
|  | 6 | 3.3877 | 365.98 | 0.0337 |
|  | 7 | 3.5512 | 349.13 | 0.0102 |
|  | 8 | 3.6074 | 343.69 | 0.1492 |
|  | 9 | 3.6839 | 336.56 | 0.0204 |
|  |  |  |  |  |
|  |  |  |  |  |
| **Dimer V** | 1 | 3.1143 | 398.11 | 0.0272 |
|  | 2 | 3.1879 | 388.92 | 0.0040 |
|  | 3 | 3.2400 | 382.67 | 0.0446 |
|  | 4 | 3.3574 | 369.29 | 0.0457 |
|  | 5 | 3.4177 | 362.77 | 0.0820 |
|  | 6 | 3.5004 | 354.20 | 0.0623 |
|  | 7 | 3.5223 | 351.99 | 0.1750 |
|  | 8 | 3.5906 | 345.31 | 0.2093 |
|  | 9 | 3.5950 | 344.88 | 0.0605 |
|  |  |  |  |  |
| **Dimer VI** | 1 | 3.1642 | 391.84 | 0.0015 |
|  | 2 | 3.1664 | 391.56 | 0.0535 |
|  | 3 | 3.2695 | 379.22 | 0.0682 |
|  | 4 | 3.2841 | 377.52 | 0.0221 |
|  | 5 | 3.4629 | 358.03 | 0.0006 |
|  | 6 | 3.4906 | 355.19 | 0.0195 |
|  | 7 | 3.5599 | 348.28 | 0.0462 |
|  | 8 | 3.6172 | 342.77 | 0.0427 |
|  | 9 | 3.6234 | 342.17 | 0.5783 |
|  |  |  |  |  |
| **Dimer VII** | 1 | 3.1926 | 388.35 | 0.0069 |
|  | 2 | 3.2096 | 386.29 | 0.0134 |
|  | 3 | 3.2971 | 376.04 | 0.0009 |
|  | 4 | 3.3107 | 374.49 | 0.0228 |
|  | 5 | 3.3161 | 373.89 | 0.0135 |
|  | 6 | 3.4403 | 360.39 | 0.0814 |
|  | 7 | 3.4824 | 356.03 | 0.0001 |
|  | 8 | 3.5419 | 350.05 | 0.0165 |
|  | 9 | 3.5826 | 346.08 | 0.0006 |
|  |  |  |  |  |

**Table S2.** Calculated E*_HOMO_* and E*_LUMO_* levels, energy gap (ΔE*_H-L_*) and dipole moment of different **CA** conformations obtained by the TD-DFT methods at B3LYP/6–31G(2d.2p) levels of theory.

| **CA Conformations** | **E*_HOMO_***  **(eV)** | **E*_LUMO_***  **(eV)** | **Δ[E*_H_-_L_*]**  **(eV)** | Experimental  **E_g_**  **(eV)** |
| --- | --- | --- | --- | --- |
| **Monomer** | -6.26 | -2.44 | 3.82 | 3.22 |
| **Dimer I** | -6.08 | -2.62 | 3.47 | 2.90* |
| **Dimer II** | -6.17 | -2.48 | 3.69 | N/A |
| **Dimer III** | -6.08 | -2.54 | 3.55 | 3.01** |
| **Dimer IV** | -6.02 | -2.40 | 3.62 | N/A |
| **Dimer V** | -6.10 | -2.42 | 3.68 | N/A |
| **Dimer VI** | -6.17 | -2.48 | 3.69 | N/A |
| **Dimer VII** | -6.19 | -2.42 | 3.77 | N/A |

*in water, **in EtOH/water (1:1.v/v)


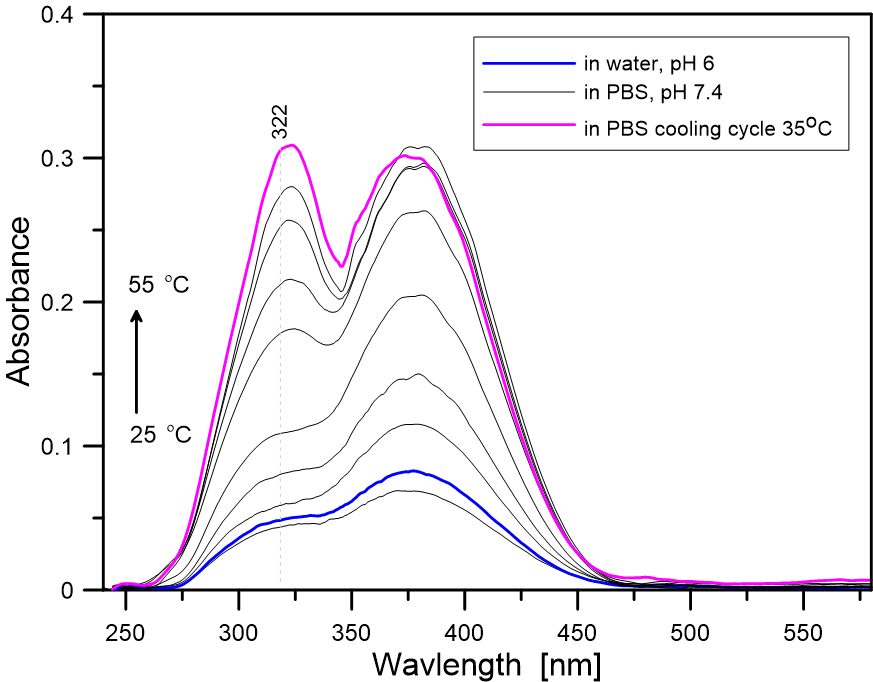


Figure S1. UV−Vis spectroscopic evaluations of CA (1.33 × 10^−5^ M) in PBS buffer occurring upon a temperature jump. All spectra were recorded immediately after temperature stabilization (<1 min).

**
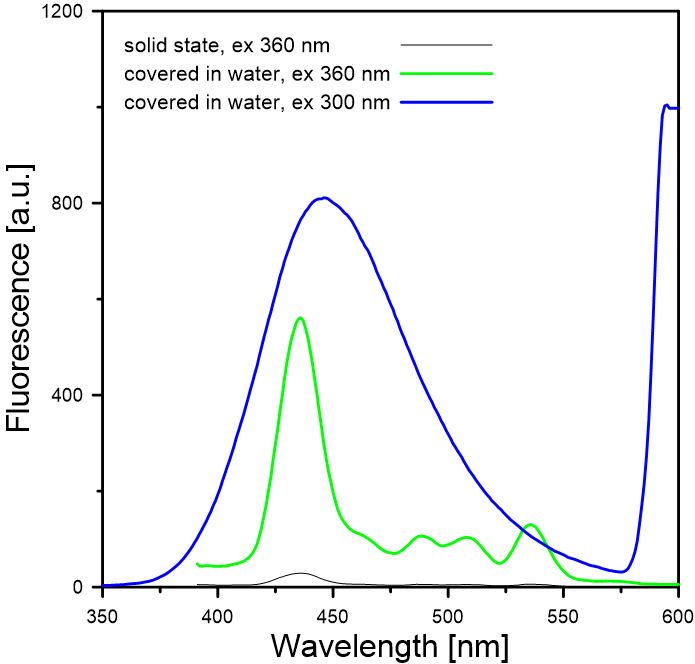
**

Figure S2. Emission spectra of **CA** in the solid state (~0.2 mg) and after adding 3 mL of water under excitation at 360 nm and 300 nm.
